# Identification of reliable reference genes for quantitative real‐time PCR analysis of the *Rhus chinensis* Mill. leaf response to temperature changes

**DOI:** 10.1002/2211-5463.13275

**Published:** 2021-09-15

**Authors:** Yanchao Chen, Biao Luo, Chuwei Liu, Zhengfeng Zhang, Chi Zhou, Ting Zhou, Guoping Peng, Xujun Wang, Waichin Li, Chuan Wu, Liqun Rao, Qiming Wang

**Affiliations:** ^1^ College of Bioscience and Biotechnology Hunan Agricultural University Changsha China; ^2^ Hunan Engineering Laboratory for Good Agricultural Practice and Comprehensive Utilization of Famous‐Region Medicinal Plants Changsha China; ^3^ Hunan Academy of Forestry Changsha China; ^4^ Department of Science and Environmental Studies The Education University of Hong Kong Tai Po China; ^5^ School of Metallurgy and Environment Central South University Changsha China

**Keywords:** RNA‐seq, reference gene, Rhus chinensis Mill., qRT‐PCR, stable expression, temperature stress

## Abstract

*Rhus chinensis* Mill. (RCM) is the host plant of *Galla chinensis*, which is valued in traditional medicine. Environmental temperature directly determines the probability of gallnut formation and RCM growth. At present, there is no experiment to systematically analyse the stability of internal reference gene (RG) expression in RCM. In this experiment, leaves that did not form gallnuts were used as the control group, while leaves that formed gallnuts were used as the experimental group. First, we conducted transcriptome experiments on RCM leaves to obtain 45 103 differential genes and functional enrichment annotations between the two groups. On this basis, this experiment established a transcriptional gene change model of leaves in the process of gallnut formation after being bitten by aphids, and RCM reference candidate genes were screened from RNA sequencing (RNA‐seq) data. This study is based on RCM transcriptome data and evaluates the stability of 11 potential reference genes under cold stress (4 °C) and heat stress (34 °C), using three statistical algorithms (geNorm, NormFinder, and BestKeeper). The results show that *GAPDH1 + PP2A2/UBQ* are stable reference genes under heat stress, while *GAPDH1 + ACT* are the most stable under cold stress. This study is the first to screen candidate reference genes in RCM and could help guide future molecular studies in this genus.

## Graphical abstract

Appropriate and stable reference genes are important for normalization of gene expression. This study is based on *Rhus chinensis* Mill. transcriptome data and evaluates the stability of 11 potential reference genes under cold stress (4 °C) and heat stress (34 °C), using three statistical algorithms (geNorm, NormFinder, and BestKeeper). This study is the first to screen candidate reference genes in *Rhus chinensis* Mill. and could help guide future molecular studies in this genus.

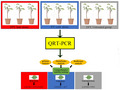

**Abbreviations**
RCM: *Rhus chinensis* Mill
qRT‐PCR: quantitative real‐time PCR
UBQ: ubiquitin
TUB1: tubulin1
TIP41: TAP42 interacting protein of 41 kDa
PP2A1: proteins phosphatase 2a subnit a1
GAPDH1: glyceraldehyde‐3‐phosphate dehydrogenase 1
ACT: actin
UPL7: E3 ubiquitin‐protein ligase 7
Ct: cycle threshold
Cp: crossing point
CV: coefficient of variation
RGs: reference genes

Quantitative real‐time PCR (qRT‐PCR) is one of the most accurate and commonly used methods to detect mRNA abundance and gene expression and is characterized by high technical sensitivity and precision. The introduction of RT‐PCR technology has greatly improved and simplified the quantification of nucleic acids and has become a valuable tool for many different disciplines [1]. Reference gene selection and sample preparation are the key issues that define the reliability of obtained qRT‐PCR data [2]. According to existing research, many reference genes (RGs) show unstable expression in different experimental organisms, experimental materials, tissues, and organs, following stress treatment, or at different stages of growth and development [3, 4]. Therefore, it is necessary to experimentally test genes that are traditionally thought to be stably expressed under different conditions and experimental treatments.

*Rhus chinensis* Mill. (RCM), also known as sumac, belongs to the *Anacardiaceae* family and has a long history of culinary use and as a traditional medicine. Asian practitioners of folk medicine have been using sumac bark for a long time, in addition to its leaves, roots, stems, fruit, and especially the galls on the leaves. Studies have shown that sumac tree compounds have strong antiviral, antibacterial, anticancer, antidiarrhoea, and antioxidant activities [5]. *Galla chinensis* is a gall formed by the parasitic aphid *Schlechtendalia chinensis* on RCM leaves, that stimulates the proliferation and expansion of leaf tissue cells. *Galla chinensis* is considered to have preventive and therapeutic effects against diseases such as diarrhoea, dysentery, rectal and bowel cancer, diabetes, septicaemia, and oral diseases. Galls on RCM leaves are rich in gallotannin (50–70%), a type of hydrolysable tannin. The global annual demand for tannins in different industrial areas, including leather processing, textile printing, and mineral separation, exceeds 15 000 tons. The total annual value of tannin related industries is approximately $30 billion [6]. Gallnut formation involves changes in the expression of many genes, which lead to major changes in hormone signalling pathways and secondary metabolite accumulation that result in the accumulation of tannins [7, 8]. Gallnut is an insect gall on RCM leaves, that is mainly parasitized by *Melaphis chinensis*, and the specific mechanism and influence of temperature on its formation have not been published. However, fruit growers in the artificial planting and harvesting base in Cili County have made long‐term observations on the effect of temperature on the formation rate of gallnuts, and this information can be shared with us. We can confirm that temperature has an important effect on gallnut formation, and temperature also affects gene expression and ultimately affects plant‐insect interactions and product formation. In addition to the interaction between aphids and RCM, temperature has an important effect on gene expression [9, 10]; however, evaluation of the stability of RCM RG expression at different temperatures has not been reported, which hampers research on the changes in gene expression related to gallnut formation and tannin accumulation.

Therefore, in view of the important role of temperature in gallnut formation, the value of gallnut as a traditional medicine and the value of tannins, which are the main components of gallnut, in industry, and other fields, it is extremely important to research the products formed by the interaction between plants and insects. The optimum selection of stable RGs under temperature stress is necessary for follow up qRT‐PCR experiments. In this study, we screened the expression of 11 candidate RGs: (Ubiquitin (*UBQ*), Tubulin1 (*TUB1*), *TUB2*, Tap interacting protein of 41 kDa (*TIP41*), proteins phosphatase 2A subnit A1 (*PP2A1*), *PP2A2*, glyceraldehyde‐3‐phosphate dehydrogenase 1 (*GAPDH1*), *GAPDH2*, *actin* (*ACT*), *actin1* (*ACT1*), and E3 ubiquitin‐protein ligase UPL7 (*UPL7*) [11, 12] for stability under temperature stress.

## Materials and methods

### Materials

RCM saplings as the materials are from the Gallnut Breeding Base in Cili County in China. Thirty saplings were transplanted from the field to pots and cultured at 24 °C and 60% humidity for 30 days. After growing more than 5 true leaves, 9 saplings with the same growth trend were selected and subjected to stress treatment using an artificial climate box. The plants were treated with the following stresses: (a) cold treatment, placed in a light incubator at 4 °C long‐days (16 h light, 8 h darkness); (b) heat stress, placed in a light incubator at 34 °C for long‐days (16 h of light, 8 h of darkness); and (c) the control group was grown for long‐days (16 h light, 8 h darkness) in a culture chamber at 24 °C. Leaves were harvested from control plants and plants treated under each stress condition for 3, 6 or 24 h. The leaves of three saplings from each treatment were blended as biological replicates, and three samples were taken altogether (Fig. S3). The samples were quickly frozen in liquid nitrogen and stored at −80 °C for RNA extraction and subsequent experiments.

### Methods

#### Total RNA extraction and cDNA synthesis

Total RNA was extracted from all samples using the Tiangen RNA prep Pure Plant Plus Kit plant (Tiangen, China) according to the manufacturer’s instructions, The RNA concentration was measured using a NanoDrop 2000, and the RNA integrity was assayed by agarose gel electrophoresis (Fig. S4). Only RNA samples with an OD260/280 ratio between 1.8 and 2.2 and an OD260/230 ratio greater than 2.0 that showed three discrete bands of 28S, 18S, and 5S were used for cDNA synthesis. Synthesis of cDNA was performed using the NovoScript Plus All‐in‐one First‐Strand cDNA Synthesis SuperMix (Novoprotein, China). According to the manufacturer’s instructions, genomic DNA was directly removed by adding gDNA Purge. The reverse transcription system of each sample was as follows: RNA template (1 μg), gDNA Purge (1 μL), Supermix (10 μL) and RNase Free Water (up to 20 μL) at 50 °C for 30 min and 75 °C for 5 min.

#### Primer design and qPCR

The primer sequences for *ACT1* were the same as those used previously [8]; the primers for all other reference genes (RGs) were designed according to transcriptome sequencing data from our group (unpublished data). First, based on the transcriptome data, common RGs such as *GAPDH* were searched, and then, the genes with the highest read count and FPKM (the highest expression) and lowest absolute value of the log2FC (the highest stability) were compared with *Arabidopsis thaliana* to determine the homology and identified as RCM reference bases. The sequences of these RGs are shown in Table S1. The annotations for these genes were compared with those in the TAIR Arabidopsis thaliana and NCBI databases. Specific primers were designed by SnapGene, and the specificity was analysed by NCBI primer blast. The primers were synthesized by Qingke (Beijing, China). The details of the genes and primers are shown in Table 1. The specificity of each primer pair was evaluated by amplification and dissolution curve analysis. The correlation coefficient (*R^2^*) and amplification efficiency (E) of the primer pairs were evaluated using standard curves for mixed cDNAs that were diluted fivefold (1/5, 1/25, 1/125, 1/625 and 1/3,125).

**Table 1:** Primer and related information for the 11 candidate RGs for quantitative qRT‐PCR analysis.

Gene Symbol	Description	Arabidopsis thaliana homology	Primer Sequence (5’–3‘)	Product length	PCR efficiency	*R^2^*
*ACT*	ACT	AT5G09810.1	F: TGTTCCCTGGTATTGCCGAC R: TGGACCAGACTCGTCGTACT	188	1.125	0.997
*ACT1*	RCM actin	AT5G09810.1	F: CATCACTCATCGGTATGGAAGC R: AGTGATTTCCTTGCTCATACGGT	164	1.008	0.999
*GAPDH1*	Glyceraldehyde‐3‐phosphate	AT3G04120.1	F: CGTGTTCCTACCGTCGATGT R: TCCTTGATGGCGGCTTTGAT	92	0.971	0.999
*GAPDH2*	Glyceraldehyde‐3‐phosphate	AT1G12900.5	F: TCCCCCTTGGATGTCATTGC R: GCGGTCAGAGACAACCTTGA	165	1.034	0.999
*PP2A1*	Pp2aa2	AT3G25800.3	F: GTCTTCTCCACCACCGACTG R: TCTGCTTGCCCCTGTTATGG	159	0.864	0.997
*PP2A2*	Protein phosphatase 2A‐4	AT3G58500.1	F: CCCTGTGACAATTTGTGGCG R: TAAGGGCCACTAACAGCGTG	161	1.101	0.999
*TUB1*	Tubulin beta‐1	AT5G23860.2	F: ACACCGAAGGAGCTGAGTTG R: CACCTCCCAAAGAGTGGCAT	108	1.106	0.993
*TUB2*	Tubulin alpha	AT1G50010.1	F: CCAACAGTGCATTTGAGCCC R: TGGGCACCAGTCAACAAACT	170	1.037	0.995
*TIP41*	Tip41	AT4G34270.1	F: ATAGGGTTTCCACTGCCACC R: GTCATGCCAAGTGGTTGGTTC	116	1.108	0.995
*UPL7*	Ubiquitin‐protein ligase 7	AT3G53090.2	F: AGCAGGTGTGAATAGGTGGC R: GTCACAGGCAGGGGTTAGTT	177	0.920	0.972
*UBQ*	Ubiquitin 11	AT4G05050.2	F: GGGTCCTCCCATCCTCAAGT R: AACTCTCCACCTCGTCCTCC	200	0.959	0.999

The qPCR instrument (Roche LightCycler 480) used 384 well blocks. Each 20 μL reaction included 10.0 μL 2×NovoStart SYBR qPCR SuperMix Plus (Novoprotein, China), 0.6 μL of each primer (10 μm), 2.0 μL diluted (1 : 10) cDNA template, and 6.8 μL RNase free water. The programme for product amplification was 94 °C for 3 min followed by 45 cycles of 94 °C for 15 s and 60 °C for 35 s. The melting curve was generated after 45 cycles. Three technical replicates were performed for each sample. The steps of the melting curve were set at 95 °C for 5 s (ramp rate: 4.80) and 65 °C for 1 min (ramp rate: 2.50).

### Data analysis

The amplification efficiency was calculated by E = 10 ^(−1/k)^‐1, where *K* is the slope of the standard curve [13]. The 2 ^−∆∆Ct^ method was applied to calculate the relative expression level of the target gene, and significant difference analysis was conducted with the spss statistical software v23.0.

MIQE checklist in Table S4.

### Algorithm to perform

geNorm was used to screen RGs, and eventually, 2 or more combinations of RGs were selected from the results computed [14]. The *M* value determines the stability of each RG. The smaller the *M* value, the higher the stability. First, ΔCp (∆Cp ≥ 0) was calculated by subtracting the smallest Cp of all the samples from the *C*p values of the other samples. Then, the 2^−∆Cp^ was calculated and used as input data to obtain the *M* and V values.

The data input required by NormFinder is the same as that required by geNorm [15], which was processed to obtain the 2^−∆Cp^ value for the RGs. The stability value of candidate genes was obtained, and it was determined that a candidate gene with a smaller stability value was more suitable to be used as an RG.

In BestKeeper, the *C*p value is directly input into the algorithm, and the correlation coefficient (R), standard deviation (SD), and coefficient of variation (CV) are obtained after the algorithm is used [16].

The stability of gene expression was evaluated with geNorm, NormFinder, and BestKeeper.

## Results

### Specificity evaluation and PCR efficiency analysis of primer amplification

In this study, 11 candidate RGs were identified based on RCM transcriptome data. The candidates were screened by analysing the genome data and gene annotation of the related species *Camelina sativa*, and alignment analysis of the Arabidopsis thaliana genes from TAIR (http://www.arabidopsis.org). Table 1 lists information for the genes, including the correlation description, primer sequence, gene number of the Arabidopsis homologue, amplicon length, PCR amplification efficiency, and correlation coefficient (*R^2^*). The amplicons generated by qRT‐PCR ranged from 92–200 bp. The specificity of the primers was confirmed by the presence of a single peak in the dissolution curve. The qRT‐PCR dissolution curve peaks for the 11 RGs are shown in Fig. S1.

**Fig. 1:**
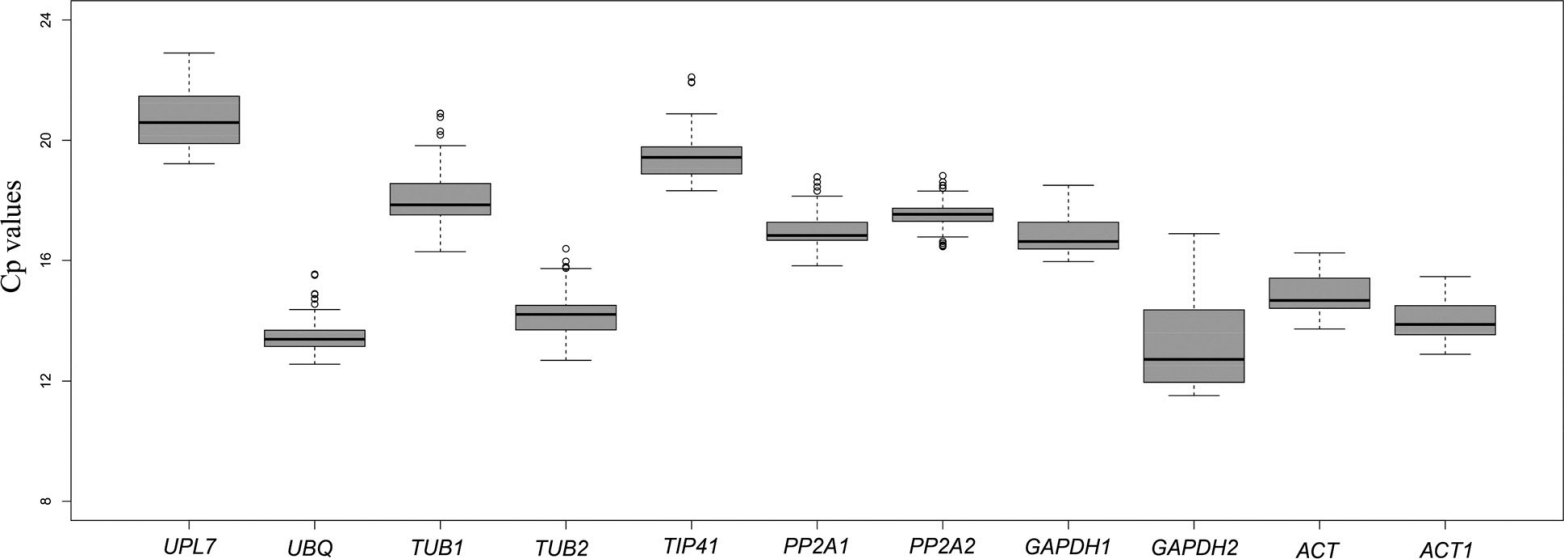
Distribution of the Cp values of the 11 candidate RGs across all samples in qRT‐PCR analysis. Boxplot analysis of crossing point (Cp) values of all samples. The boxes represent the interquartile range. The line across the box represents the median. Hyphens over and under the boxes are respectively shown as the maximum and minimum.

Fig. 1 shows the crossing point (Cp) values for all samples (*n* = 33). The candidate gene with the highest abundance among all RGs was *GAPDH2*, which had the lowest mean Cp value of 13.34 ± 1.55 (mean ± SD) and thus the highest expression. In contrast, *UPL7* (20.7 ± 0.971) had the highest mean Cp value (lowest expression). In addition, *PP2A2* showed low variability and the lowest SD value (SD = 0.567) and might therefore be the most stably expressed RG in all samples, but further algorithms are required to support this hypothesis. Although the expression level of *GAPDH2* (SD = 1.55) showed the most variability, and the SD was greater than 1, its unstable expression as an RG could reduce the accuracy of the results. Fig. S2 provides the separate Cp results of these genes for the control, heat, and cold treatments with respect to the time points (3, 6 and 24 h). To further evaluate the stability of the expression of the 11 genes in different samples exposed to different stresses, different algorithms were used to analyse the data.

### Expression stability analysis of candidate reference genes

To further evaluate the stability of the expression of different RGs in response to different temperature stresses using other tools, the qRT‐PCR data Cp values were corrected and preprocessed into appropriate forms that could be recognized and analysed by three different algorithms (geNorm, NormFinder and BestKeeper). The use of geNorm and NormFinder for analysis necessitated conversion of the Cp values obtained by qRT‐PCR into the relative gene expression. For this, the lowest Cp value in all samples was identified and then subtracted from the Cp values of the other samples. As a result, the ∆Cp value was ≥ 0. The ∆Cp value of each gene in each sample was obtained. The ∆Cp value of 2^−∆cp^ of the corresponding genes in the corresponding sample was calculated, which represents the relative quantitative data for each candidate RG, and comprises the data used for analysis by geNorm and NormFinder. BestKeeper does not require conversion of the Cp value obtained by RT‐PCR, but can use the Cp value directly [17].

### geNorm analysis

**Fig. 2:**
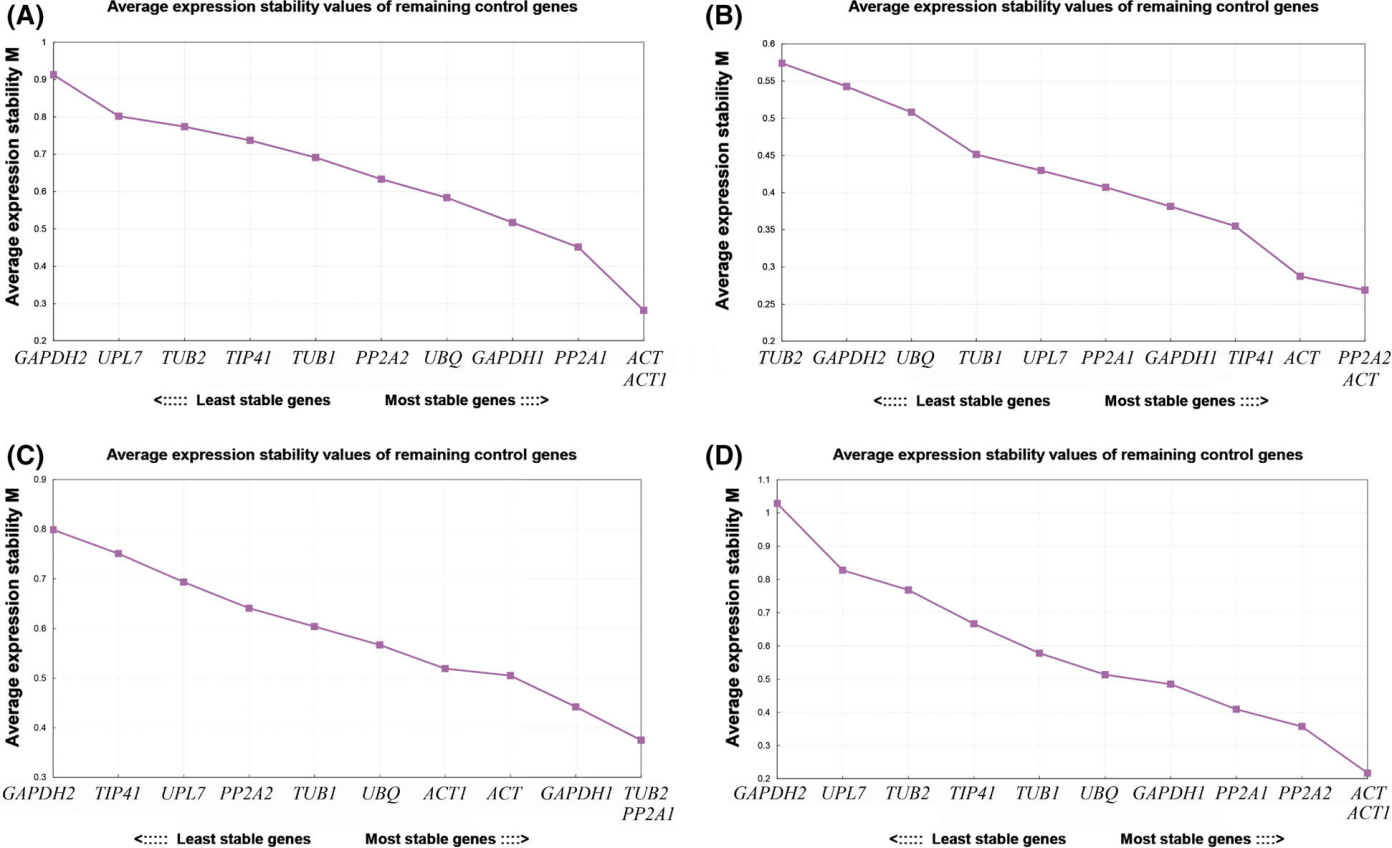
Average expression stability values (*M*) of 11 candidate RGs by geNorm analyse: (A) all samples; (B) control group untreated; (C) under cold stress; (D) under heat stress.

**Fig. 3:**
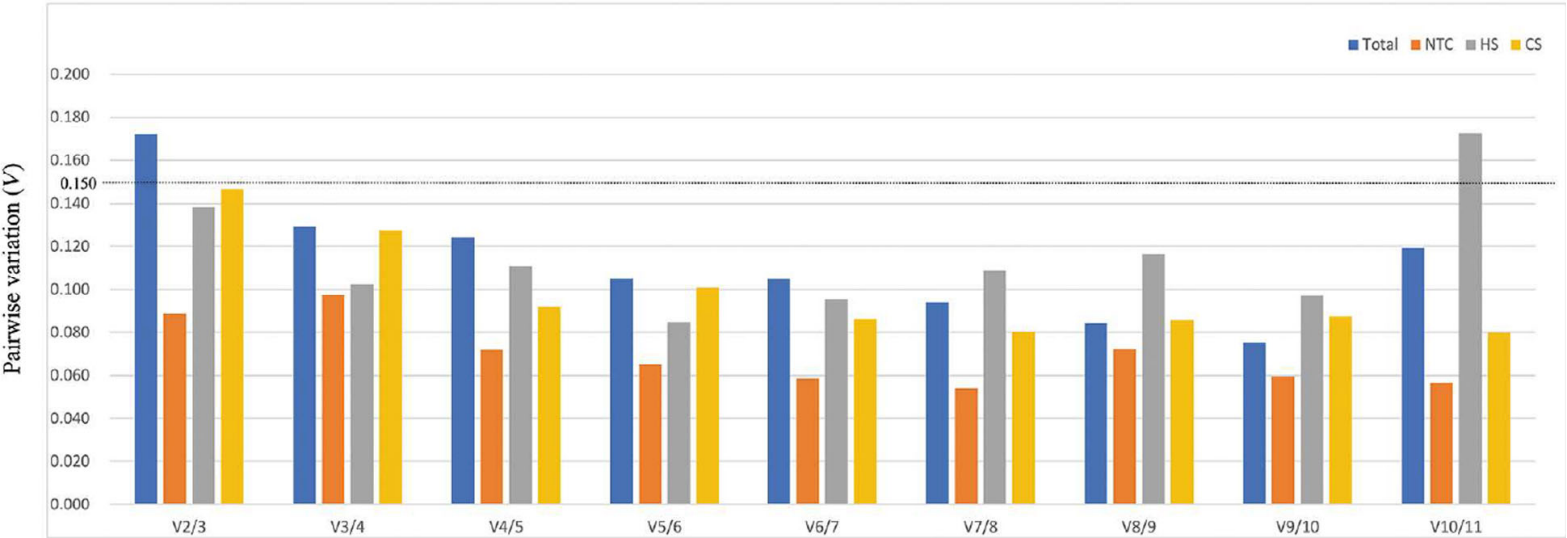
Determination of best RGs number by geNorm pairwise variation (Vn/Vn+1). (Total) all samples; (NTC) control group untreated; (CS) under cold stress; (HS) under heat stress.

The whole dataset was divided into the following four groups: all samples group, cold stress sample group, heat stress sample group, and untreated control sample group. The results showed that the stability of the 11 RGs differed among these groups (Fig. 2). For example, *GAPDH2* and *UPL7* showed poor stability under various treatments and controls. *ACT*, *ACT1,* and *PP2A2* were more stably expressed than other RGs in the control group (Fig. 2B) and in the heat stressed group (Fig. 2D). In the cold treated group, *TUB2, GAPDH1*, and *PP2A1* showed highly stable expression (Fig. 2C). According to a Vn/Vn + 1 cut‐off value of 0.15 for pairwise variations, the first number of n RGs is sufficient for accurate normalization [16]. In this article, the V2/3 values of the samples under cold and heat stress and the control group were less than 0.15 (Fig. 3), which indicates that the two RGs are sufficient for accurate normalization of all the samples. The Vn/n + 1 values of all samples were less than 0.15, except for V2/3 of the all‐sample group and V10/11 of the heat stress group (Table S3), indicating that accurate normalization and reliable results can be obtained by using the n value corresponding to the number of RGs.

### NormFinder analysis

Based on the results of the NormFinder analysis, Table 2 shows the expression stability and ranking of all of the candidate RGs. For the all‐sample group, *GAPDH1* was the most stably expressed gene and in the control, low‐ and high‐temperature groups, the stability of *GAPDH1* was similar to that obtained by geNorm analysis. *GAPDH1* (0.134), *ACT* (0.188), and *TIP41* (0.185) were highly stable in the control group (Table 2); *GAPDH1* (0.069), *PP2A2* (0.094), and *UBQ* (0.229) were stable in the heat‐treated group; and *GAPDH1* (0.159), *TUB2* (0.185), and *PP2A1* (0.248) were stable in the cold treated group. Notably, *GAPDH2* showed the lowest expression stability in all the treatment groups, indicating that it is not a stable RG.

**Table 2:** Stability analysis of candidate RGs, as assayed with NormFinder software. NTC represents the no‐treatment control group. CS represents under cold stress. HS represents under heat stress.

Rank	Total	Stability value	NTC	Stability value	Hs	Stability value	Cs	Stability value
1	*GAPDH1*	0.133	*GAPDH1*	0.134	*GAPDH1*	0.069	*GAPDH1*	0.159
2	*ACT*	0.286	*TIP41*	0.185	*PP2A2*	0.094	*TUB2*	0.185
3	*ACT1*	0.329	*ACT*	0.188	*UBQ*	0.229	*PP2A1*	0.248
4	*UBQ*	0.347	*TUB1*	0.249	*ACT1*	0.329	*ACT*	0.313
5	*PP2A1*	0.361	*ACT1*	0.251	*PP2A1*	0.363	*UBQ*	0.314
6	*TUB1*	0.444	*PP2A2*	0.266	*ACT*	0.369	*ACT1*	0.377
7	*TIP41*	0.462	*UPL7*	0.296	*TUB1*	0.380	*TUB1*	0.405
8	*TUB2*	0.463	*PP2A1*	0.299	*TIP41*	0.521	*PP2A2*	0.477
9	*PP2A2*	0.488	*UBQ*	0.377	*TUB2*	0.668	*UPL7*	0.504
10	*UPL7*	0.530	*TUB2*	0.394	*UPL7*	0.718	*TIP41*	0.593
11	*GAPDH2*	0.912	*GAPDH2*	0.430	*GAPDH2*	1.347	*GAPDH2*	0.609

### BestKeeper analysis

Based on the results of the BestKeeper analysis, in the cold treated group, *ACT* (2.91 ± 0.44), *ACT1* (2.93 ± 0.42), and *GAPDH1* (3.11 ± 0.54) were the top RGs (Table 3). In the heat‐treated group, *UBQ* (1.1 ± 0.15), *PIP2* (1.41 ± 0.28), and *TIP41* (1.57 ± 0.31) were the top RGs. In addition, *GAPDH1* and *TIP41* were highly stable in the cold and heat*‐*treated groups. In contrast, the SD value of *GAPDH2* was greater than 1 in the heat*‐*treated group, and the stability ranking in the other groups was also low, indicating that its stability was clearly the worst. The BestKeeper analysis suggested that the unstable expression of an RG is indicated by SD greater than 1 [16]. Although only two RGs, *TUB1* under cold stress and *GAPDH2* under heat stress, showed SD values greater than 1 in our analysis, half of the 11 candidate genes had CV values lower than 4.

**Table 3:** Expression stability values of the 11 candidate RGs calculated using BestKeeper. NTC represents the no‐treatment control group. CS represents under cold stress. HS represents under heat stress. SD represents standard deviation. CV represents coefficient of variation.

Rank	Total	CV ± SD	NTC	CV ± SD	Cs	CV ± SD	Hs	CV ± SD
1	*PP2A2*	2.42 ± 0.42	*GAPDH1*	0.74 ± 0.12	*ACT*	2.91 ± 0.44	*UBQ*	1.10 ± 0.15
2	*TIP41*	2.74 ± 0.53	*TIP41*	1.47 ± 0.28	*ACT1*	2.93 ± 0.42	*TIP41*	1.57 ± 0.31
3	*PP2A1*	2.74 ± 0.47	*TUB1*	1.49 ± 0.26	*GAPDH1*	3.11 ± 0.54	*GAPDH1*	1.74 ± 0.29
4	*GAPDH1*	2.88 ± 0.49	*ACT*	1.62 ± 0.24	*TIP41*	3.25 ± 0.65	*PP2A1*	2.49 ± 0.41
5	*ACT1*	3.40 ± 0.48	*UPL7*	1.74 ± 0.35	*PP2A2*	3.34 ± 0.58	*PP2A2*	2.61 ± 0.47
6	*ACT*	3.49 ± 0.52	*PP2A1*	1.76 ± 0.30	*PP2A1*	3.46 ± 0.60	*UPL7*	3.52 ± 0.71
7	*UPL7*	3.59 ± 0.74	*PP2A2*	1.84 ± 0.32	*UPL7*	3.90 ± 0.83	*ACT1*	4.78 ± 0.67
8	*UBQ*	4.16 ± 0.57	*ACT1*	2.31 ± 0.32	*TUB2*	4.73 ± 0.70	*ACT*	4.92 ± 0.73
9	*TUB1*	4.69 ± 0.85	*UBQ*	2.91 ± 0.39	*TUB1*	5.63 ± 1.06	*TUB1*	5.18 ± 0.91
10	*TUB2*	4.90 ± 0.70	*TUB2*	3.71 ± 0.5	*GAPDH2*	6.05 ± 0.85	*TUB2*	5.33 ± 0.77
11	*GAPDH2*	9.64 ± 1.29	*GAPDH2*	3.91 ± 0.48	*UBQ*	6.49 ± 0.90	*GAPDH2*	12.79 ± 1.80

### Comprehensive stability ranking

The geometric means of different RG rankings are used to determine the most stably expressed RGs under different conditions [18]. Comprehensive stability ranking results and the most stable RGs are shown in Table 4. *GAPDH1* (3.00), *PP2A2* (3.33), and *UBQ* (3.33) ranked the top three in the heat stress group, while *GAPDH1*(2.33) and *ACT* (3.00) ranked among the top two in the cold stress group. The results from all the treatment groups indicate that *GAPDH1* (3.00) and *ACT* (3.00) were the most reliable RGs among the 11 candidate genes. In the control (no‐treatment) group, *TIP41* (2.67) and *GAPDH1* (2.33) were the most reliable RGs among the 11 candidate genes. *GAPDH2* showed poor stability in all the treatment groups and was therefore unsuitable for use as an RG. It has good stability of *GAPDH1* that the ranking in each result was ≤ 3.0. So *GAPDH1* + *PP2A2*/*UBQ* were recommended for use in the heat stress group, and *GAPDH1* + *ACT* were recommended for use in the cold stress group.

**Table 4:** Statistics on the ranking of 11 RGs in all statistical algorithms. NTC represents the no‐treatment control group. CS represents under cold stress. HS represents under heat stress.

Statistical algorithms	group	*ACT1*	*ACT*	*PP2A1*	*PP2A2*	*GAPDH1*	*GAPDH2*	*TIP41*	*UPL7*	*TUB1*	*TUB2*	*UBQ*
geNorm	Total Rank	2	1	3	6	4	11	8	10	7	9	5
NormFinder		3	2	5	9	1	11	7	10	6	8	4
BestKeeper		5	6	3	1	4	11	2	7	9	10	8
Average		3.33	3.00	3.67	5.33	3.00	11.00	5.67	9.00	7.33	9.00	5.67
geNorm	NTC Rank	3	2	6	1	5	10	4	7	8	11	9
NormFinder		5	3	8	6	1	11	2	7	4	10	9
BestKeeper		8	4	6	7	1	11	2	5	3	10	9
Average		5.33	3.00	6.67	4.67	2.33	10.67	2.67	6.33	5.00	10.33	9.00
geNorm	HS Rank	1	2	4	3	5	11	8	10	7	9	6
NormFinder		4	6	5	2	1	11	8	10	7	9	3
BestKeeper		7	8	4	5	3	11	2	6	9	10	1
Average		4.00	5.33	4.33	3.33	3.00	11.00	6.00	8.67	7.67	9.33	3.33
geNorm	CS Rank	5	4	2	8	3	11	10	9	7	1	6
NormFinder		6	4	3	8	1	11	10	9	7	2	5
BestKeeper		2	1	6	5	3	10	4	7	9	8	11
Average		4.33	3.00	3.67	7.00	2.33	10.67	8.00	8.33	7.67	3.67	7.33

### Validation of the selected reference genes

**Fig. 4:**
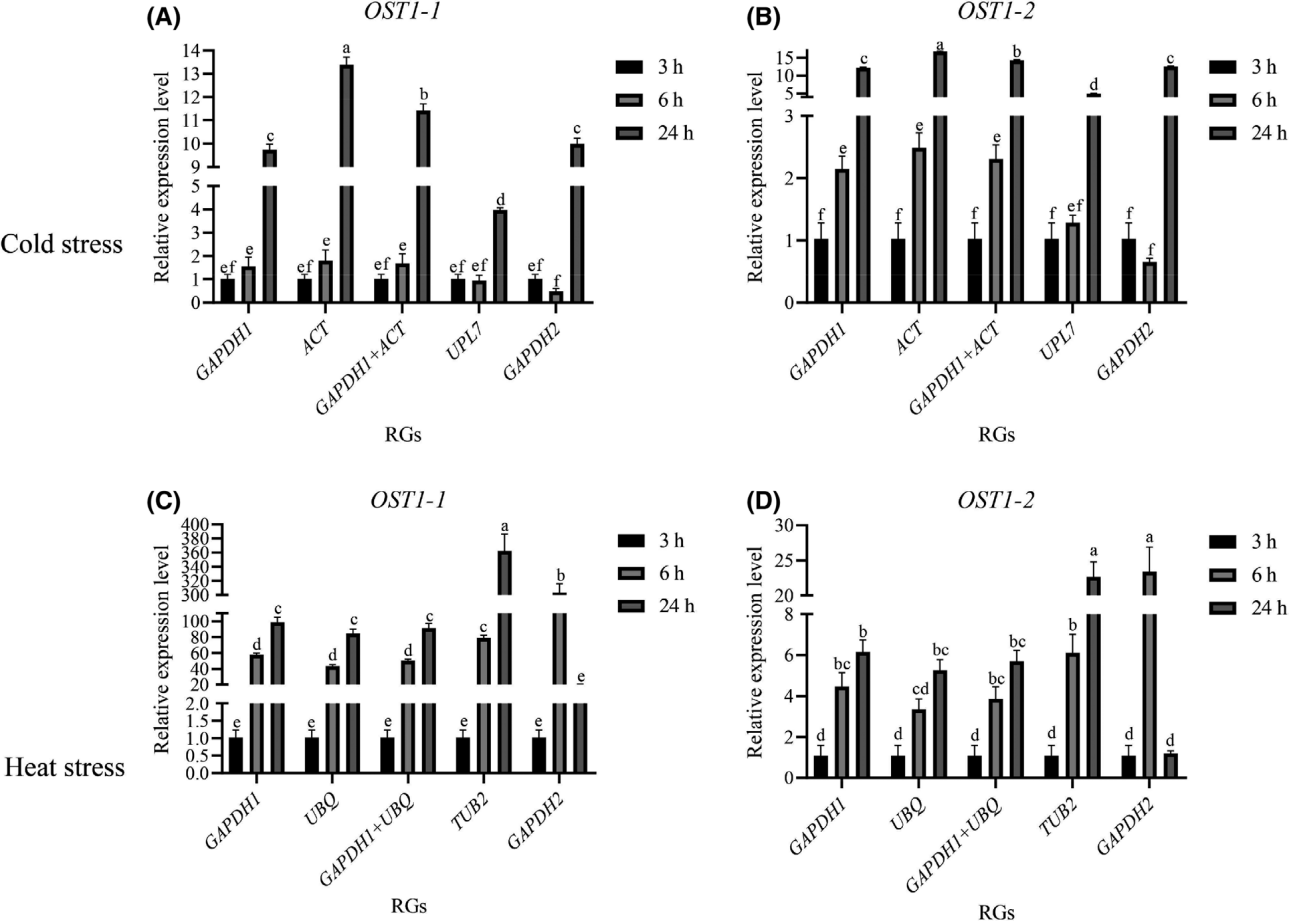
The relative expression level of target gene *OST1‐1* and *OST1‐2* in different experimental conditions and RGs. (A) (B) under cold stress(C) (D) under heat stress. Error bars indicate standard deviation. Data are mean ± SD and analysed by one‐way ANOVA. Different letters above the bars represent significant differences (*P* < 0.05).

To validate the stability of the potential RGs selected using different algorithms and evaluation methods, the relative expression levels of *OST1‐1* and *OST1‐2* under temperature stress were verified, these are temperature‐related genes of interest in the RCM transcriptome [19], and changes in the stability of candidate RGs under 3, 6, and 24 h of cold and heat stress were analysed based on their relative expression levels (Fig. 4). Under cold stress, *ACT* and *GAPDH1* ranked as the most stable in the comprehensive evaluation and were used to verify *OST* after normalization by the two RGs. In Fig. 4A, the relative expression of *OST1‐1* was upregulated at 6 h compared with 3 h and was significantly upregulated at 24 h by approximately 11‐fold. The expression of *OST1‐2* was upregulated at 6 h compared with 3 h and was significantly upregulated at 24 h by approximately 13‐fold (Fig. 4B). Under heat stress, *GAPDH1* and *UBQ* were selected as RGs with stable expression for verification. Although the average comprehensive ranking of *UBQ* and *PP2A2* was the same under heat stress, the highest ranking of *UBQ* in the three algorithms was higher than that of *PP2A2*, so two pairs of RGs, *UBQ* and *GAPDH1*, were selected for verification. The expression levels of *OST1‐1* were upregulated by approximately 40 times and 80 times at 6 h and 24 h (Fig. 4C), respectively. The expression levels of *OST1‐*2 were upregulated approximately 4‐ and 6‐fold at 6 h and 24 h (Fig. 4D), respectively. When *GAPDH2* with the lowest stability ranking was used as an RG to verify the relative expression level of the *OST*, the results obtained were completely opposite to the relative expression level of other candidate genes as an RG at 6 h, which was precisely caused by the instability of *GAPDH2* itself. This result confirms the importance of the stability of RGs for experimental expression measurements under different conditions.

## Discussion

Although the RCM genome has not been sequenced, RCM plants possess important medicinal and industrial value, which is mainly due to their high tannin contents [7, 20, 21]. Environmental temperature is a critical factor for RCM growth and gallnut formation. Gallnuts are usually collected from RCMs planted on the shady side of mountains. The main goal of identifying stably expressed RGs in response to temperature in RCM is to reveal the basis of gallnut formation in relation to temperature. Previous research has not addressed the stability of RG expression. Several classic and commonly used RGs are often selected for the relative quantification of gene expression by qRT‐PCR in plant or animal materials under various experimental conditions [22]. In recent years, in addition to the study of stable RG expression in many different plants, model plants have also been used to evaluate RGs under certain conditions [23, 24]. It is increasingly important to systematically select and evaluate the stability of RGs [25], which will contribute to more accurate quantification of gene expression under different experimental conditions and for different research purposes.

The stability of internal RGs according to geNorm analysis is indicated by the size of the *M* value; however, this value does not give an accurate threshold above which the internal RGs are unstable. The most stable RGs can only be selected as those with the lowest *M* value. Here, the *M* value for 11 candidate RGs was less than 0.8 under cold stress and less than 1.1 under heat stress, but the difference between the most stable and most unstable *M* value was 0.80 for heat stress treatments and only 0.39 for cold stress treatments. This suggests that the *M* value of the most unstable RG under cold stress was twice as high as that of the most stable RG, whereas that under heat stress was approximately 4‐fold higher. A similar result was obtained from analysis using NormFinder, which also did not provide an accurate threshold with which to estimate the cut‐off between candidate gene stability and instability.

In our study, among the 11 candidate RGs, 4 pairs of RGs (*ACT1*/*ACT2*, *GAPDH1*/*GAPDH2*, *PP2A1*/*PP2A2*, and *TUB1*/*TUB2*) belonged to the same gene families but showed great differences in stability. As an example, the two candidate genes with the same name as *GAPDH* showed relatively large stability differences, which was also found in other studies [26]. Whether these differences are caused by the genes themselves or the differences caused by the specific amplified fragments that affect the amplification experiment is worth further study.

According to a survey, 20 plant species have been used to study RGs in recent years, and these studies involve the 11 candidate genes identified in this paper (Table S2). The 43 internal RGs in the summary table ( Table S2 [3, 4, 11, 12, 27, 28, 29, 30, 31, 32, 33, 34, 35, 36, 37, 38, 39, 40, 41, 42]) are reported to be highly stably expressed under various stress conditions and sampling tissues, and the four RGs with the highest frequency of occurrence are *EF1α*, *ACT*, *GAPDH,* and *PP2A*. However, under abnormal temperature conditions, the 43 RGs showed similar behaviours in 19 plant species, for example, *EF1α*, *TUB*, *PP2A,* and *GAPDH* were highly stable in response to cold stress, and notably, under heat stress. Thus, the stability of homologous genes in different species is not consistent.

## Conclusions

In this study, the recommended RGs under heat and cold stresses were similar to those recommended for 19 plant species. It is generally recommended to use multiple RGs to normalize expression data for most plant materials to improve accuracy. Because it is difficult to select appropriate RGs for each individual study, the statistics for different RGs here provide a good resource for gene expression analysis. Because differences in the expression of key genes are subtle, it is extremely important that RG expression stably responds to treatment. Therefore, this research provides information that is useful for the verification of gene expression in RCM under temperature stress and is useful for future research on gallnut formation. The screening and evaluation of the stability of the expression of 11 candidate RGs in RCM under temperature stress identified *GAPDH1*+ *PP2A2*/*UBQ* as the most suitable RG under heat stress, while *GAPDH1*+*ACT* are the most suitable under cold stress. In the untreated group, *GAPDH1*+*TIP41*, which are RGs, are recommended in general. In all treatment groups, *GAPDH1*+*ACT* were the recommended RGs. The results provide key technical guidance for the further study of RCMs under different temperature stresses and may also suggest candidate RGs for studies with other plant species.

## Conflict of interest

The authors declare no conflict of interest.

## Author contribution

QW and LR analysed the results and conceived the project. YC and QW designed the experiments and wrote the paper. YC performed the experiment. XW provided the plant material. ZZ, CL and CZ carried out the experiments and analysed the data. TZ, BL prepared the expematerials. GP, WL and CW discussed the results, commented on the manuscript, and reviewed the article. All authors have read and approved the final manuscript.

## Supporting information

**Fig. S1**. Distribution of the Cp values of the 11 candidate RGs across all samples in qRT‐PCR analysis. Boxplot analysis of crossing point (Cp) values of all samples. The boxes represent the interquartile range. The line across the box represents the median. Hyphens over and under the boxes are respectively shown as the maximum and minimum.

**Fig. S2**. Average expression stability values (*M*) of 11 candidate RGs by geNorm analyse: (A) all samples; (B) control group untreated; (C) under cold stress; (D) under heat stress.

**Fig. S3**. Determination of best RGs number by geNorm pairwise variation (Vn/Vn+1). (Total) all samples; (NTC) control group untreated; (CS) under cold stress; (HS) under heat stress.

**Fig. S4**. The relative expression level of target gene *OST1‐1* and *OST1‐2* in different experimental conditions and RGs. (A) (B) under cold stress(C) (D) under heat stress. Error bars indicate standard deviation. Data are mean ± SD and analysed by one‐way ANOVA. Different letters above the bars represent significant differences (*P* < 0.05).

**Table S1**. Sequence of RGs.
**Table S3**. Determination of best RG number calculated by geNorm pairwise variation (Vn/Vn + 1): Keep the value to three decimal places
**Table S4**. MIQE checklist.

**Table S2**. Summary and statistical table of related research on various plant materials in recent years.

## Data Availability

All the data are available from the corresponding author upon request.
