# Supplementary figures and images for: Identification of reliable reference genes for quantitative real‐time PCR analysis of the Rhus chinensis Mill. leaf response to temperature changes
Source: FEBS Open Bio. 2021 Sep 15;11(10):2763–73. doi: 10.1002/2211-5463.13275 (PMC8487043; doi:10.1002/2211-5463.13275)

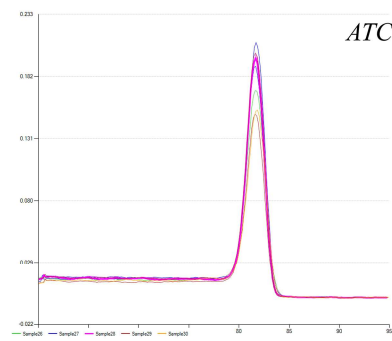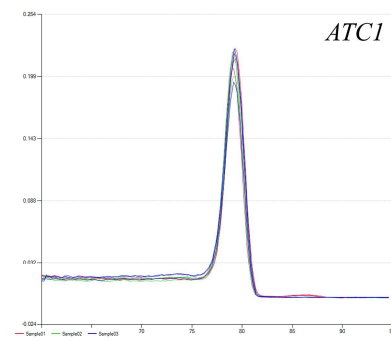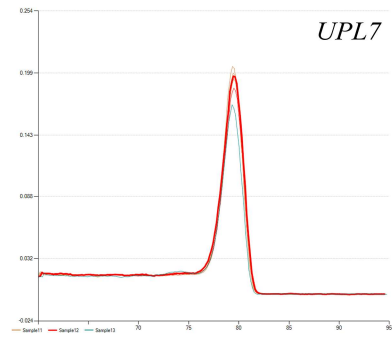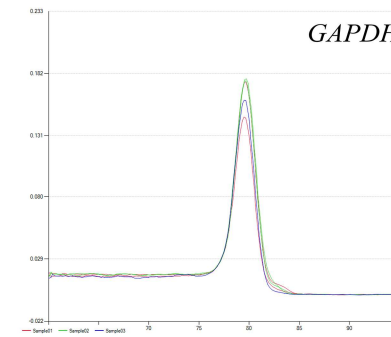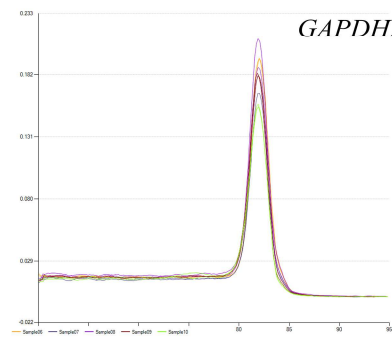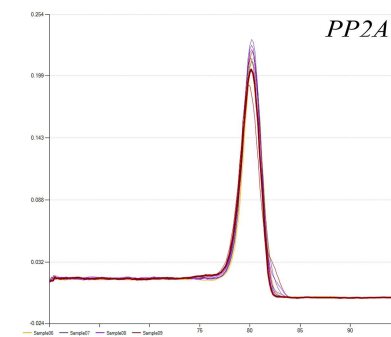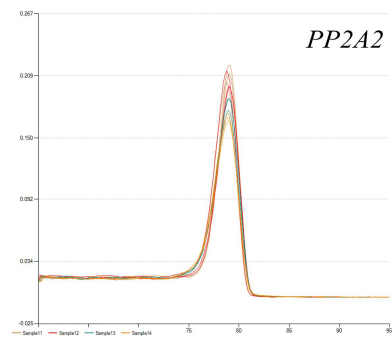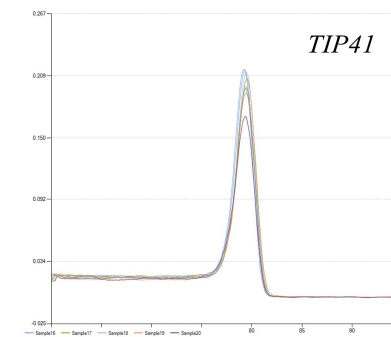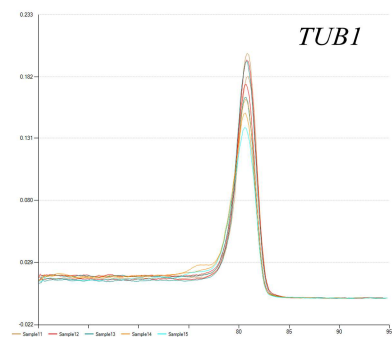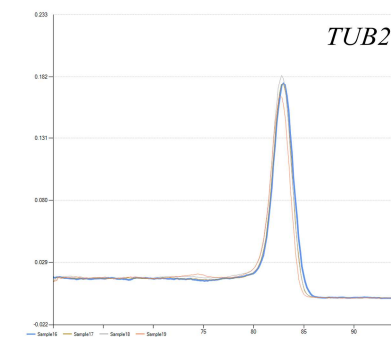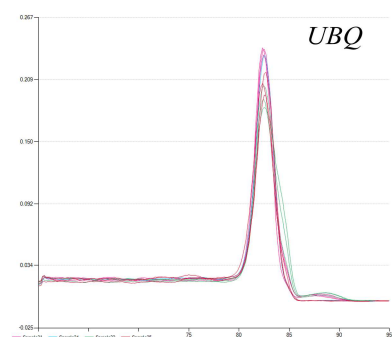

Supplement: Supplementary file 1 — Fig. S1. Distribution of the Cp values of the 11 candidate RGs across all samples in qRT‐PCR analysis. Boxplot analysis of crossing point (Cp) values of all samples. The boxes represent the interquartile range. The line across the box represents the median. Hyphens over and under the boxes are respectively shown as the maximum and minimum. [file FEB4-11-2763-s006.pdf]

*ACT*

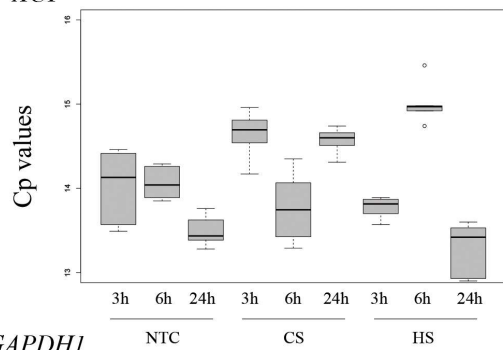

*ACT1*

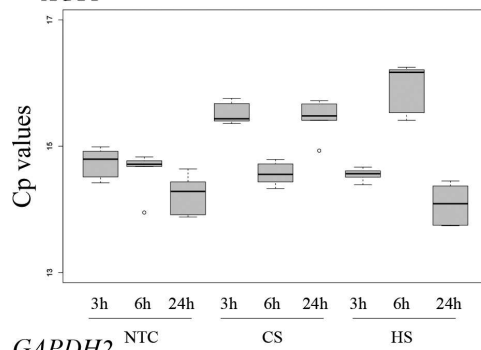

*TIP41*

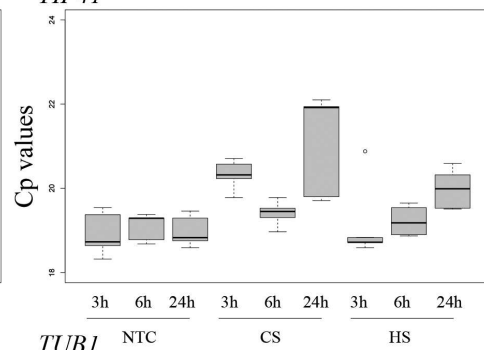

*GAPDH1*

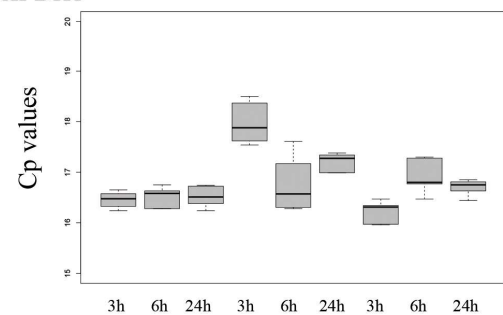

*GAPDH2*

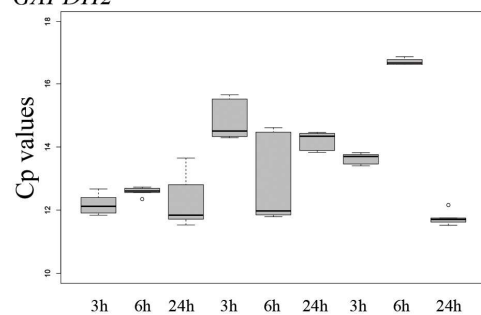

*TUB1*

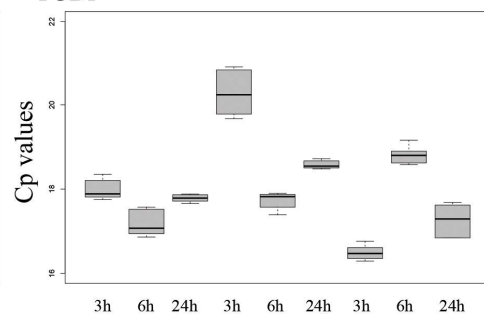

*PP2A1*

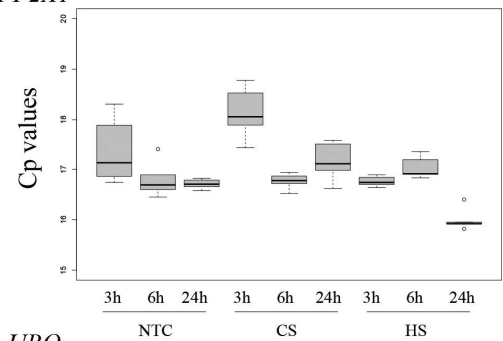

*PP2A2*

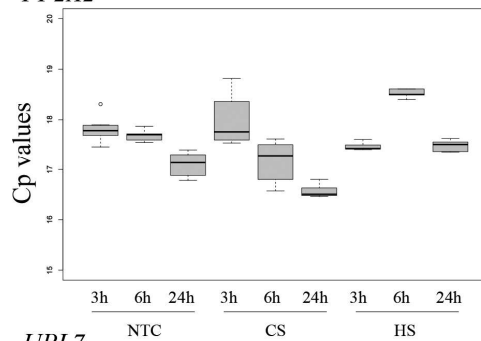

*TUB2*

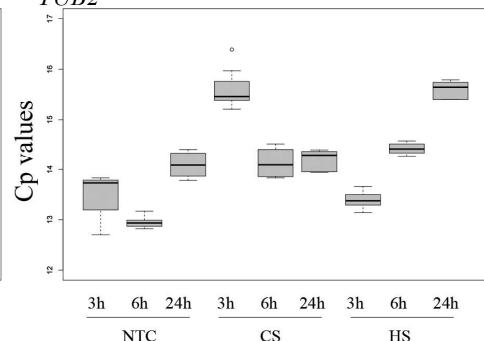

*UBQ*

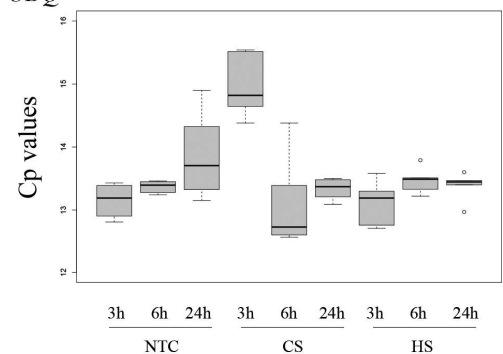

*UPL7*

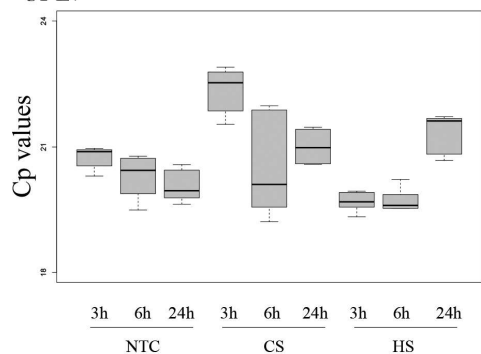

Supplement: Supplementary file 2 — Fig. S2. Average expression stability values (M) of 11 candidate RGs by geNorm analyse: (A) all samples; (B) control group untreated; (C) under cold stress; (D) under heat stress. [file FEB4-11-2763-s003.pdf]

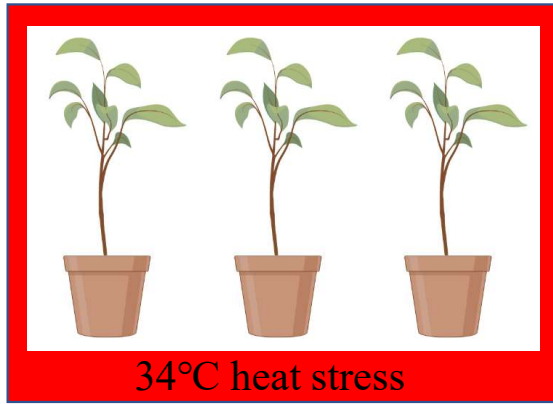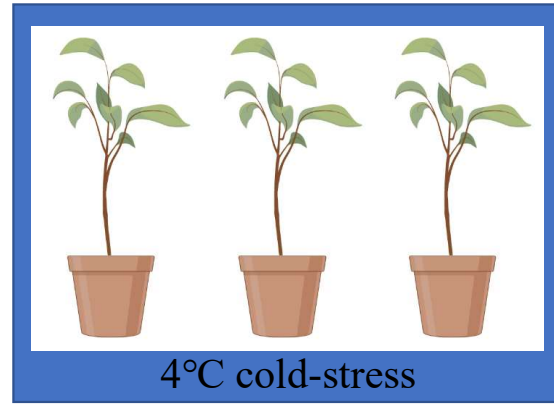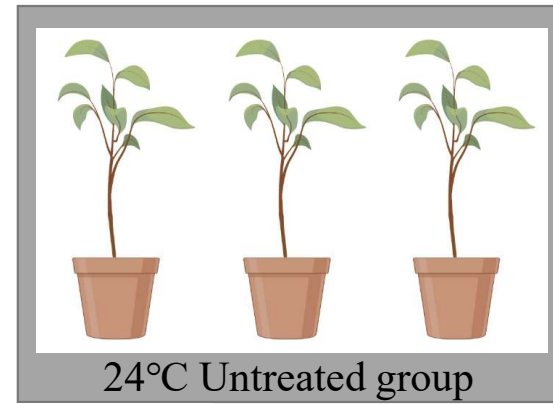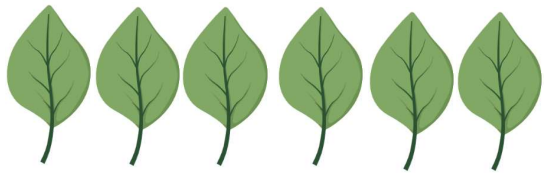

A lot of leaves mix each group

About 100 mg Each tube

---

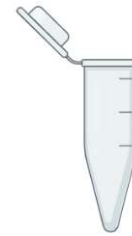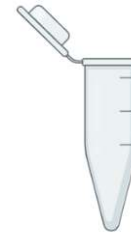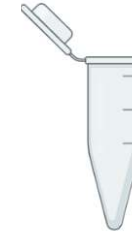

heat stress

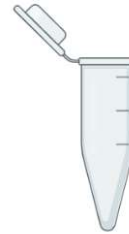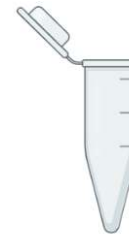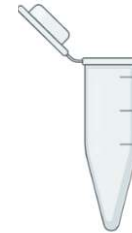

cold-stress

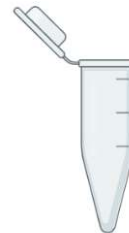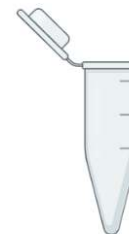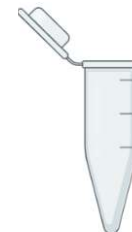

Untreated group

Supplement: Supplementary file 3 — Fig. S3. Determination of best RGs number by geNorm pairwise variation (Vn/Vn+1). (Total) all samples; (NTC) control group untreated; (CS) under cold stress; (HS) under heat stress. [file FEB4-11-2763-s005.pdf]
