# Supplementary material for: Identification of reliable reference genes for quantitative real‐time PCR analysis of the Rhus chinensis Mill. leaf response to temperature changes
Source: FEBS Open Bio. 2021 Sep 15;11(10):2763–73. doi: 10.1002/2211-5463.13275 (PMC8487043; doi:10.1002/2211-5463.13275)
Supplement: Supplementary file 4 — Fig. S4. The relative expression level of target gene OST1‐1 and OST1‐2 in different experimental conditions and RGs. (A) (B) under cold stress(C) (D) under heat stress. Error bars indicate standard deviation. Data are mean ± SD and analysed by one‐way ANOVA. Different letters above the bars represent significant differences (P < 0.05). [file FEB4-11-2763-s001.pdf]

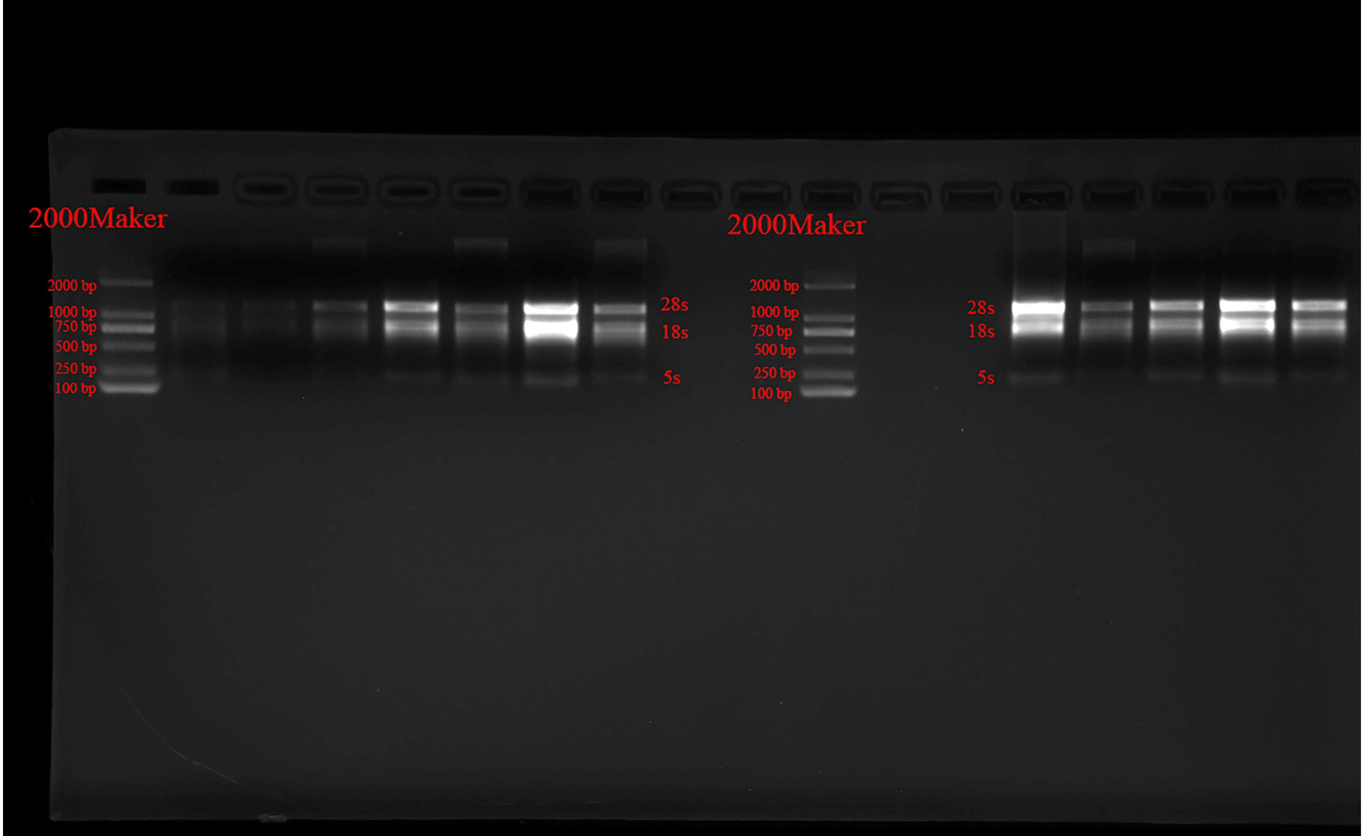
2000Maker
2000 bp
1000 bp
750 bp
500 bp
250 bp
100 bp
28s
18s
5s
2000Maker
2000 bp
1000 bp
750 bp
500 bp
250 bp
100 bp
28s
18s
5s

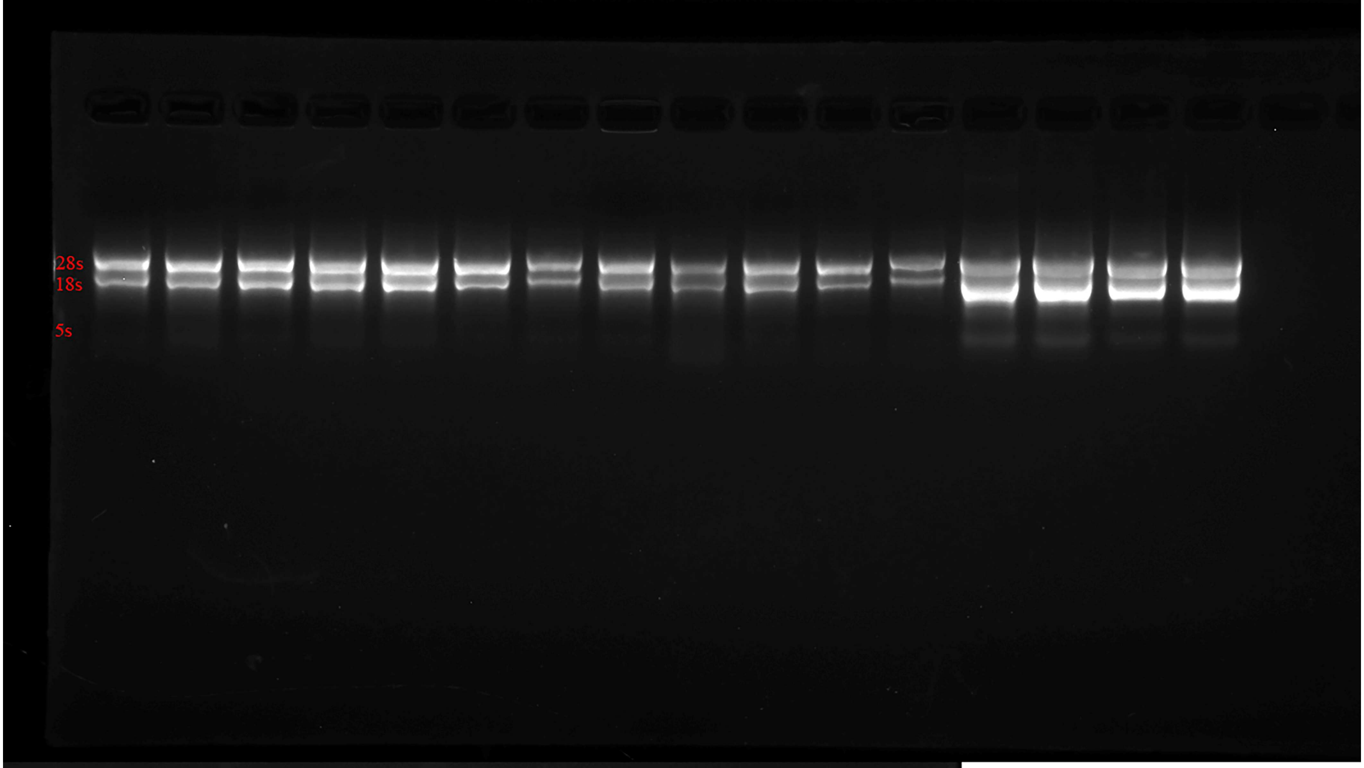
28s
18s
5s

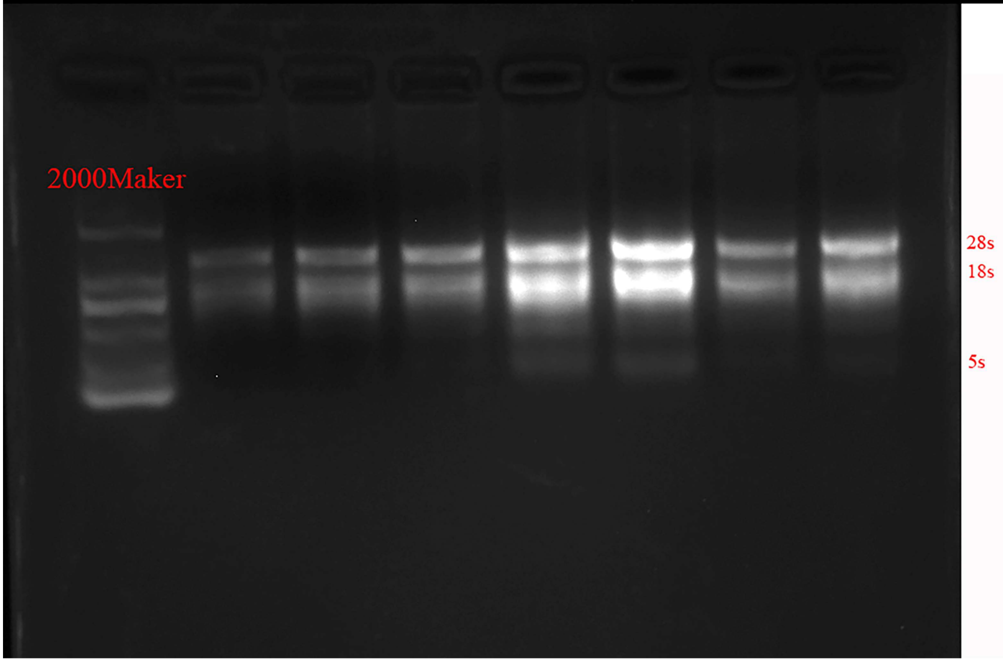
2000Maker
28s
18s
5s
