# Supplementary material for: Identification of reliable reference genes for quantitative real‐time PCR analysis of the Rhus chinensis Mill. leaf response to temperature changes
Source: FEBS Open Bio. 2021 Sep 15;11(10):2763–73. doi: 10.1002/2211-5463.13275 (PMC8487043; doi:10.1002/2211-5463.13275)
Supplement: Supplementary file 5 — Table S1. Sequence of RGs. Table S3. Determination of best RG number calculated by geNorm pairwise variation (Vn/Vn + 1): Keep the value to three decimal places Table S4. MIQE checklist. [file FEB4-11-2763-s002.docx]

Table S1. Sequence of RGs

| Gene and RNA-Seq ID | Amplification sequences |
| --- | --- |
| ***UPL7***  Cluster-13816.36156 | >AGCAGGTGTGAATAGGTGGCACAGAACAGATGAAGCAGACATGACATATCTTTTGAAATTCCTGGTGGCCCACATTTCAAAGGTTTTACATCCCAGACATCAGAAGATTCCTCATCCACCTGTACTGTCTTAGGTCGACCATCAACATTATCAGTATAACTAACCCCTGCCTGTGAC |
| ***UBQ***  Cluster-13816.54529 | >GGGTCCTCCCATCCTCAAGTTGCTTTCCTGCAAAAATCAGACGCTGCTGGTCTGGTGGGATCCCCTCCTTGTCTTGGATCTTTGCCTTAACGTTGTCGATGGTGTCGGAGCTCTCCACCTCAAGAGTTATGGTCTTTCCGGTGAGGGTTTTGACGAAAATCTGCATGCCTCCACGGAGACGGAGGACGAGGTGGAGAGTT |
| ***TUB1***  Cluster-13816.48370 | >ACACCGAAGGAGCTGAGTTGATCGACTCCGTTCTCGATGTTGTCAGGAAAGAGGCTGAGAACTGTGACTGCTTGCAAGGTTTCCAAGTATGCCACTCTTTGGGAGGTG |
| ***TUB2***  Cluster-13816.55421 | >CCAACAGTGCATTTGAGCCCTCATCTATGATGGCCAAATGTGACCCCCGCCATGGCAAGTACATGGCTTGCTGCCTCATGTACCGTGGTGATGTTGTGCCAAAGGATGTCAATGCTGCTGTTGCTACCATCAAGACCAAGCGTACCATTCAGTTTGTTGACTGGTGCCCA |
| ***TIP41***  Cluster-13816.65377 | >ATAGGGTTTCCACTGCCACCAAAAGCACAATGCATCCTGGTGTCCCTCAATCTCATAAGTACTCCATCAACTCTAAGCCAAAATCGCAAGAGAAGGAACCAACCACTTGGCATGAC |
| ***PP2A1***  Cluster-13816.52010 | >GTCTTCTCCACCACCGACTGGTCTACAATCGGAATGAGAGACTGCAGCACCTTTGCAACATTAAACTTTATGTTGGGTACTCTGTCTTTTGATGCATGGACGACCACTGGCAACAATTTAGAACAAGTGATTTCTGGTCCCATAACAGGGGCAAGCAGA |
| ***PP2A2***  Cluster-13816.61266 | >CCCTGTGACAATTTGTGGCGATATTCATGGGCAGTTTCATGATCTTGCAGAACTTTTTCAAATTGGAGGGAAGTGTCCAGATACCAACTACTTGTTTATGGGAGATTATGTGGACCGTGGTTATTACTCTGTTGAAACTGTCACGCTGTTAGTGGCCCTTA |
| ***GAPDH1***  Cluster-13816.55683 | >CGTGTTCCTACCGTCGATGTTTCAGTGGTAGACCTCACTGTCAGACTTGAGAAGGGGGCAACCTATGATGAAATCAAAGCCGCCATCAAGGA |
| ***GAPDH2***  Cluster-13816.56501 | >TCCCCCTTGGATGTCATTGCCATCAATGACACCGGAGGTGTCAAGCAGGCCTCCCACCTTCTCAAATACGATTCCACTCTTGGCATCTTTGATGCCGATGTCAAACCTGTTGGTGACAATGCCATCTCCGTAGACGGAAAGGTCATCAAGGTTGTCTCTGACCGC |
| ***ACT***  Cluster-13816.54879 | >TGTTCCCTGGTATTGCCGACCGTATGAGCAAGGAAATCACTGCACTTGCTCCAAGCAGCATGAAGATCAAGGTGGTTGCTCCACCAGAGAGAAAATACAGTGTCTGGATCGGAGGATCAATCCTTGCATCCCTCAGCACCTTCCAGCAGATGTGGATTTCAAAGGGTGAGTACGACGAGTCTGGTCCA |
| ***ACT1***  RCM actin | >CATCACTCATCGGTATGGAAGCTGCTGGTATCCATGAGACCACCTACAACTCCATCATGAAGTGTGATGTTGATATCAGAAAGGATCTCTATGGTAACATTGTGCTTAGTGGTGGTTCAACTATGTTCCCTGGTATTGCCGACCGTATGAGCAAGGAAATCACT |
| ***OST1-1***  Cluster-13816.62641 | >AAAGCAGCTCGCAAATCAGCTGATGTTCAGTTGAAAGAGCTTTCAAAGGAGTTGAAGCCCCTGTTGGCATTCTTGCAATCTTCATCAGCCGCCTCC |
| ***OST1-2***  Cluster-13816.49876 | >CTCGCGACACCTGTACTCTCCGATCTGATCCTCTCCAAAGTCGATCGACGCATTGATTTGACTTCACAAATTGTTCGCAGCGTTTCAACACTTAAGGTGGAGAATGCTGGCCCTGAGTT |

The yellow part are the primers for the verification of the target genes

**Table S3.** Determination of best RG number calculated by geNorm pairwise variation (Vn/Vn + 1): Keep the value to three decimal places

|  | **V2/3** | **V3/4** | **V4/5** | **V5/6** | **V6/7** | **V7/8** | **V8/9** | **V9/10** | **V10/11** |
| --- | --- | --- | --- | --- | --- | --- | --- | --- | --- |
| **Total** | 0.172 | 0.129 | 0.124 | 0.105 | 0.105 | 0.094 | 0.084 | 0.075 | 0.119 |
| **NTC** | 0.089 | 0.098 | 0.072 | 0.065 | 0.059 | 0.054 | 0.072 | 0.060 | 0.057 |
| **HS** | 0.138 | 0.102 | 0.111 | 0.085 | 0.095 | 0.109 | 0.116 | 0.097 | 0.173 |
| **CS** | 0.147 | 0.127 | 0.092 | 0.101 | 0.086 | 0.080 | 0.086 | 0.087 | 0.080 |

NTC represents the no treatment control group. CS represents under cold stress. HS represents under heat stress.

**Table S4.** MIQE checklist for authors, reviewers, and editors.

| Experimental design | | qPCR oligonucleotides | |
| --- | --- | --- | --- |
| Definition of experimental and control groups | In paper materials and methods | Primer sequences | Table1 in paper |
| Number within each group |  | Complete reaction conditions | The qPCR instrument (Roche LightCycler 480) used 384-well blocks. Each 20-μL reaction included 10.0 μL 2×NovoStart SYBR qPCR SuperMix Plus (Novoprotein, Chi-na), 0.6 μL each primer (10μM), 2.0 μL diluted (1:10) cDNA template, and 6.8 μL RNase-free water. The program for product amplification was 94°C for 3 min followed by 45 cycles of 94°C for 15 s and 60°C for 35 s. The melting curve was generated after 45 cycles. Three technical replicates were performed for each sample. The steps of the melting curve were set 95℃ 5 seconds (ramp rate:4.80).65℃ 1min (ramp rate:2.50). |
| Sample Description | RCM leafs | Reaction volume and amount of cDNA/DNA |  |
| Volume/mass of sample processed | The fresh weight of about 100 mg | Primer concentrations |  |
| Processing procedure | In paper materials and methods | Buffer/kit identity and manufacturer |  |
| Sample storage conditions and duration | quickly frozen in liquid nitrogen and stored at -80°C | Additives (SYBR Green I, DMSO, and so forth) |  |
| Nucleic acid extraction | RNA extract | Manufacturer of plates/tubes and catalog number | SYBR Green |
| Name of kit and details of any modifications | Tiangen RNA prep Pure Plant Plus Kit plant (Tiangen, China) | Manufacturer of qPCR instrument | Roche LightCycler 480  384 384-well blocks |
| Details of DNase or RNase treatment | In one step of the centrifuge column method, DNase is added for removal | qPCR validation | Roche LightCycler 480 |
| Contamination assessment (DNA or RNA) | OD260/280 ratio between 1.8 and 2.2 | Specificity (gel, sequence, melt, or digest) | melting curve Fig S1 |
| Nucleic acid quantification | NanoDrop 2000 | PCR efficiency calculated from slope | Table1 in paper |
| Purity ( A 260 / A 280) | between 1.8 and 2.2 | Data analysi | TBtools，EXCEL |
| RNA integrity: method/instrument | agarose gel electrophoresis three discrete bands of 28S, 18S and 5S | qPCR analysis program (source, version) |  |
| Electrophoresis traces | In fig | Justification of number and choice of reference genes | this paper mainly studies |
| Reverse transcription | | statistical methods for results significance  Software (source, version) | SPSS.23.0  Single factor ANVOA test |
| Complete reaction conditions | 42°C 5min  50°C 15min  75°C 5min |  |  |
| Amount of RNA and reaction volume | RNA template 1μg  Volume:20μl |  |  |
| Manufacturer of reagents and catalogue numbers | NovoScript Plus All-in-one 1st Strand cDNA Synthesis SuperMix, |  |  |
| Storage conditions of cDNA | short period -20°C  long period -70°C |  |  |
| qPCR target information | |  |  |
| Gene symbol | Table1 in paper |  |  |
| Sequence accession number |  |  |  |
| Amplicon length |  |  |  |
